# Whole-transcriptome sequencing identified gene expression signatures associated with aggressive clear cell renal cell carcinoma

**DOI:** 10.18632/genesandcancer.183

**Published:** 2018-05

**Authors:** Ken Batai, Elliot Imler, Jayce Pangilinan, Robert Bell, Aye Lwin, Elinora Price, Tijana Milinic, Amit Arora, Nathan A. Ellis, Erika Bracamonte, Bruce Seligmann, Benjamin R. Lee

**Affiliations:** ^1^ Division of Urology, Department of Surgery, University of Arizona, Tucson, AZ, USA; ^2^ BioSpyder Technologies, Inc, Tucson, AZ, USA; ^3^ Department of Pathology, University of Arizona, Tucson, AZ, USA; ^4^ Department of Surgery, University of Arizona, Tucson, AZ, USA; ^5^ Department of Epidemiology and Biostatistics, University of Arizona, Tucson, AZ, USA; ^6^ Department of Cellular and Molecular Medicine, University of Arizona, Tucson, AZ, USA

**Keywords:** kidney cancer, biomarkers, molecular subtype, Hispanic Americans, whole transcriptome sequencing

## Abstract

Clear cell renal cell carcinoma (ccRCC) is the most prevalent subtype of kidney cancer, yet molecular biomarkers have not been used for the prognosis of ccRCC to aide clinical decision making. This study aimed to identify genes associated with ccRCC aggressiveness and overall survival (OS). Samples of ccRCC tumor tissue were obtained from 33 patients who underwent nephrectomy. Gene expression was determined using whole-transcriptome sequencing. The Cancer Genome Atlas Kidney Renal Clear Cell Carcinoma (TCGA-KIRC) RNA-seq data was used to test association with OS. 290 genes were differentially expressed between tumors with high and low stage, size, grade, and necrosis (SSIGN) score (≥7 *vs*. ≤3) with *P*_ADJ_<0.05. Four genes, *G6PD*, *APLP1*, *GCNT3*, and *PLPP2*, were also over-expressed in advanced stage (III and IV) and high grade (3 and 4) ccRCC and tumor with necrosis (*P*_ADJ_<0.05). Investigation stratifying by stage found that *APLP1* and *PLPP2* overexpression were significantly associated with poorer OS in the early stage (Quartile 1 vs. Quartile 4, HR = 3.87, 95% CI:1.25-11.97, *P* = 0.02 and HR = 4.77, 95% CI:1.37-16.57, *P* = 0.04 respectively). These genes are potential biomarkers of ccRCC aggressiveness and prognosis that direct clinical and surgical management.

## INTRODUCTION

Kidney cancer is among the 10 most common cancers [1], and both globally and nationally, its incidence has significantly increased over the past 30 years since the 1980s [2]. Currently, prognosis of renal cell carcinoma (RCC) and post-surgical surveillance strategies are mainly based on staging and histology. A composite score combining multiple factors including stage, size, grade, and necrosis (SSIGN) score may be useful for predicting prognosis [3, 4], but molecular biomarkers prognostic of local recurrence also helps develop individually-tailored clinical management strategies before or after nephrectomy.

Several gene expression studies have identified molecular subtypes within RCC, and within the most common subtype of RCC, clear cell renal carcinoma (ccRCC) [5, 6]. Patients with ccRCC molecular subtype ccB (CC-e.3) have worse survival rates than patients with subtype ccA (CC-e.1 and CC-e.2) [5, 6]. Another study demonstrated that molecular subtypes are correlated with SSIGN score [7]. Gene expression studies also identified some genes that predict ccRCC aggressiveness and progression [8–11]. However, currently there is no verified set of molecular biomarkers that can be used in a clinical setting to move toward precision medicine of RCC treatment, much less such a signature measured from formalin-fixed paraffin embedded (FFPE) on a platform that can be used clinically. Moreover, Hispanic Americans (HAs) and Native American have been found to have a higher burden of kidney cancer, but they are often underrepresented in the molecular studies [12, 13]. Including the high risk previously underrepresented populations may help identify novel molecular signatures.

In this study, we measured gene expression profiles of ccRCC directly from FFPE cores to identify molecular subtypes and gene expression signatures that were associated with ccRCC aggressiveness as assessed by tumor characteristics, including the SSIGN score. A novel extraction-free whole transcriptome sequencing method (TempO-Seq™) was used to measure gene expression. Then, The Cancer Genome Atlas Kidney Renal Clear Cell Carcinoma (TCGA-KIRC) RNA-seq data was used to verify the findings and test association between identified gene expression signatures and overall survival (OS).

## RESULTS

### Patients' characteristics

Table 1 shows that characteristics of 33 patients included in this study. The mean age of patients was 56.7, and about half of patients had advanced stage ccRCC (54.5%) with 7 patients (21.2%) who had metastasis at presentation. Necrosis was found in tumor from 15 patients (48%). While 14 patients (42.4%) had low SSIGN score, 12 patients (36.4%) had high SSIGN score. We observed 6 recurrences and 4 deaths during the follow-up period (median follow-up days of 908 for recurrence and 932 for mortality). Of 4 patients who died, 3 died from disease specific cause and one died from complication related to surgery. Of 33 patients, 15 were EAs and 15 were HAs. HA patients and patients from unknown racial/ethnic background were younger than EA patients (mean age of 54.3 in HAs compared to 60.4 in EAs). Compared to HAs, EA patients had significantly higher creatinine levels (*P* = 0.01). Metastasis and high-grade tumor was slightly more common in EAs than HAs (33.3% *vs* 13.3% for metastasis at presentation and 73.3% *vs*. 66.7% for Fuhrman Grade). EAs and other racial/ethnic groups were similar in other aspects.

**Table 1:** Patients characteristics

	Total (*n* = 33)	EAs (*n* = 15)	HAs (*n* = 15)	unknown (*n* = 3)
**Age, mean (SD)**	56.7 (13.6)	60.4 (15.1)	54.3 (11.7)	49.7 (13.2)
**Gender, *n* (%)**				
** Female**	15 (45.5%)	7 (46.7%)	6 (40.0%)	2 (66.7%)
** Male**	18 (54.5%)	8 (53.3%)	9 (60.0%)	1 (33.3)
**Body Mass Index, *n* (%)**				
** Normal (<30)**	19 (57.6)	9 (60.0)	9 (60.0)	1 (33.3)
** Obese (≥30)**	14 (42.4)	6 (40.0)	6 (40.0)	2 (66.7)
**Diabetes, *n* (%)**				
** No**	22 (66.7)	10 (66.7)	11 (73.3)	1 (33.3)
** Yes**	11 (33.3)	5 (33.3)	4 (26.7)	2 (66.7)
**Hypertension, *n* (%)**				
** No**	13 (39.4%)	5 (33.3%)	6 (40.0%)	2 (66.7%)
** Yes**	20 (60.6%)	10 (66.7%)	9 (60.0%)	1 (33.3%)
**Creatinine (mg/dL), Median (IQR)**	0.80 (0.80-1.00)	0.90 (0.85-1.20)	0.80 (0.70-0.90)^*^	0.80 (0.80-0.80)
**GFR (ml/min/1.73cm2), *n* (%)**				
** <60**	4 (12.9%)	2 (15.4%)	2 (13.3%)	0 (0.0%)
** >60**	27 (87.1%)	11 (84.6%)	13 (86.7%)	3 (100.0%)
**TNM Stage, *n* (%)**				
** I or II**	15 (45.8%)	7 (46.7%)	7 (46.7%)	1 (33.3)
** III or IV**	18 (54.5%)	8 (53.3%)	8 (53.3%)	2 (66.7)
**Tumor Stage, n (%)**				
** T1**	14 (42.4%)	7 (46.7%)	6 (40.0%)	1 (33.3%)
** T2**	1 (3.0%)	0 (0.0%)	1 (6.7%)	0 (0.0%)
** T3**	17 (51.5%)	7 (46.7%)	8 (53.3%)	2 (66.7%)
** T4**	1 (3.0%)	1 (6.7%)	0 (0.0%)	0 (0.0%)
**Regional Lymph Nodes, *n* (%)**				
** No**	30 (90.9%)	13 (86.7%)	14 (93.3%)	3 (100.0%)
** Yes**	3 (9.1%)	2 (13.3%)	1 (6.7%)	0 (0.0%)
**Metastasis, *n* (%)**				
** No**	26 (78.8%)	10 (66.7%)	13 (86.7%)	3 (100.0%)
** Yes**	7 (21.2%)	5 (33.3%)	2 (13.3%)	0 (0.0%)
**Fuhrman Grade, *n* (%)**				
** 1 or 2**	10 (30.3%)	4 (26.7%)	5 (33.3%)	1 (33.3%)
** 3 or 4**	23 (69.7%)	11 (73.3%)	10 (66.7%)	2 (66.7%)
**Necrosis, *n* (%)**				
** No**	16 (51.6%)	7 (50%)	7 (50%)	2 (66.7%)
** Yes**	15 (48.4%)	7 (50%)	7 (50%)	1 (33.3%)
**Tumor Size (diameter, cm), mean (SD)**	6.5 (3.9)	5.9 (3.4)	7.3 (4.6)	5.3 (1.1)
**SSIGN, *n* (%)**				
** Low (≤3)**	14 (42.4%)	7 (46.7%)	5 (33.3%)	2 (66.7%)
** Intermediate (4-6)**	7 (21.2%)	2 (13.3%)	4 (26.7%)	1 (33.3%)
** High (≥7)**	12 (36.4%)	6 (40.0%)	6 (40.0%)	0 (0.0%)
**Negative/Positive Margins, *n* (%)**				
** Positive**	9 (27.3%)	5 (33.3%)	4 (26.7%)	0 (0.0%)
** Negative**	24 (72.7%)	10 (66.7%)	11 (73.3%)	3 (100.0%)
**Recurrence, *n* (%)**				
** No**	27 (81.8%)	12 (80.0%)	12 (80.08%)	3 (100.0%)
** Yes**	6 (18.2%)	3 (20.0%)	3 (20.0%)	0 (0.0%)
**Vital Status, *n* (%)**				
** Alive**	29 (87.9%)	14 (93.3%)	12 (80.0%)	3 (100.0%)
** Deceased**	4 (12.1%)	1 (6.7%)	3 (20.0%)	0 (0.0%)

^*^ (*P* = 0.01 (Independent Sample Mann-Whitney U-Test)

### Molecular subtype

The TempO-Seq data successfully grouped 32 out of 33 patients into previously defined molecular subtypes (ccA and ccB) (Supplementary Figure 1). ccA and ccB subtype could not be assigned to one HA patient with advanced stage ccRCC. ccB patients were significantly older (*P* = 0.04) and had higher creatinine levels (*P* = 0.03) than patients with ccA subtype (Table 2). The molecular subtype ccB was found in 58.3% of patients with higher SSIGN score (≥7), while 42.9% of patients with low SSIGN score (≤3) had ccB. The subtype ccB was more common in patients with Fuhrman Grade 3 or 4 (50.0%), metastasis (57.1%), and tumor necrosis (60.0%) than patients with Furman Grade 1 or 2 (30.0%) and patients without metastasis (40.0%) and tumor necrosis (31.3%). Patients with subtype ccB also had larger tumor size than patients with subtype ccA (mean, 7.5 *vs.* 5.8 cm). ccB was more common among patients who had recurrence (66.7%) than who did not (38.5%). Moreover, all the patients who passed away had ccB subtype. Molecular subtype, ccA, was more common in HAs than EAs (64.3% *vs*. 41.2%).

**Table 2:** Molecular subtypes (ccA *vs*. ccB)

	ccA (*n* = 18)	ccB (*n* = 14)
**Age, mean (SD)^1^**	52.2 (15.8)	61.4 (7.8)
**Gender, *n* (%)**		
** Male**	7 (41.2%)	10 (58.8%)
** Female**	11 (73.3%)	4 (26.7%)
**Race/ethnicity, *n* (%)**		
** European Americans**	6 (40.0%)	9 (60.0%)
** Hispanic Americans**	9 (64.3%)	5 (35.7%)
** Others**	3 (100.0%)	
**Creatinine, Median (IQR)^2^**	0.80 (0.70-0.90)	0.95 (0.80-1.20)
**GFR, *n* (%)**		
** <60**	2 (50.0%)	2 (50.0%)
** >60**	16 (61.5%)	10 (38.5%)
**Fuhrman Grade, *n* (%)**		
** 1 or 2**	7 (70.0%)	3 (30.0%)
** 3 or 4**	11 (50.0%)	11 (50.0%)
**TNM Stage, *n* (%)**		
** I or II**	9 (60.0%)	6 (40.0%)
** III or IV**	9 (52.9%)	8 (47.1%)
**Metastasis at Presentation, *n* (%)**		
** No**	15 (60.0%)	10 (40.0%)
** Yes**	3 (42.9%)	4 (57.1%)
**Necrosis, *n* (%)**		
** No**	11 (68.8%)	5 (31.3%)
** Yes**	6 (40.0%)	9 (60.0%)
**Tumor Size, mean (SD)**	5.8 (2.9)	7.5 (4.8)
**SSIGN, *n* (%)**		
** Low (≤3)**	8 (57.1%)	6 (42.9%)
** High (≥7)**	5 (41.7%)	7 (58.3%)
**Recurrence, *n* (%)**		
** No**	16 (61.5%)	10 (38.5%)
** Yes**	2 (33.3%)	4 (66.7%)
**Vital Status, *n* (%)^3^**		
** Alive**	18 (64.3%)	10 (35.7%)
** Deceased**	0 (0.0%)	4 (100.0%)

^1^*P* = 0.04 (Independent Sample T-test)

^2^*P* = 0.03 (Independent Sample Mann-Whitney U-Test)

^3^*P* = 0.04 (Chi-Square Test)

### Differentially expressed genes between aggressive and non-aggressive ccRCC

We performed two step analysies to identify differentially expressed genes between aggressive and non-aggressive ccRCC. First, comparing patients with low *vs* high SSIGN score (≤3 *vs*. ≥7) identified 590 differentially expressed genes (*P*_ADJ_ < 0.05) (Supplementary Figure 2). 281 genes were over-expressed and 309 genes were down-regulated in the ccRCC with high SSIGN score. Then, we performed analysis to identify genes that were also differentially expressed 1) between advanced and early stage, 2) between high and low grade, and 3) between tumors with necrosis and without. Of these 590 genes, only 4 genes (*APLP1*, *G6PD*, *GCNT* 3, and *PLPP2*) were consistently differentially expressed, and they were over-expressed in advanced stage and high-grade tumors as well as tumors with necrosis (*P*_ADJ_ < 0.05) (Table 3). The most strongly over-expressed gene was amyloid beta precursor like protein 1, *APLP1*. *APLP1* had 2.8-fold higher expression in tumor with high compared to low SSIGN score (*P*_ADJ_ = 4.4×10^−6^). *APLP1* also had 2.4-fold higher expression in advanced stage ccRCC and 2.6-fold higher expression in high-grade ccRCC. However, although trending for significance, expression difference between tumor and normal samples for *APLP1* was not statistically significant after adjusting for multiple comparison (*P*_ADJ_ = 0.06), while three other genes were over-expressed in tumor compared to normal tissue samples (*P*_ADJ_ < 0.05).

**Table 3:** Genes overexpressed (Log_2_ fold change) in aggressive ccRCC

Gene	Gene Name	FC (SSIGN)	FC (Stage)	FC (Grade)	FC (Necrosis)
***APLP1***	amyloid beta precursor like protein 1	2.8	2.4	2.6	1.9
***G6PD***	glucose-6-phosphate dehydrogenase	2.5	2.0	1.6	2.8
***GCNT3***	glucosaminyl (N-acetyl) transferase 3, mucin type	2.3	2.1	2.1	2.3
***PLPP2***	phospholipid phosphatase 2	2.3	1.9	1.9	2.4

Among genes implicated in RCC, *BAP1* and *SETD2* were down regulated in aggressive ccRCC (*P* < 0.05), but other genes, *PBRM1*, *KDM5C*, *VHL*, *PTEN*, and *MTOR*, were not associated with aggressive ccRCC (Supplementary Table 1). We also evaluated differential expression between high and low SSIGN score for 64 genes associated with ccRCC aggressiveness, recurrence, or survival identified in previous studies [8, 9, 11, 18–21]. 34 genes were validated with nominal *P* < 0.05, and 19 genes, including *S1PR1*, *TSPAN7*, *EDNRB*, had *P*_ADJ_ < 0.05 (Supplementary Table 2).

We investigated if the expression of *APLP1*, *G6PD*, *GCNT*3, and *PLPP2* were correlated with other clinical characteristics and outcomes (Supplementary Table 3). Correlation between *G6PD* expression and pre-operative creatinine levels was statistically significant (Spearman's *rho* 0.423, *P* = 0.02). *APLP1* and *G6PD* were overexpressed in patients without metastatic ccRCC at presentation who had recurrence later (*P* = 0.03 and *P* = 0.01 respectively). We observed significantly higher expression of *APLP1*, *G6PD*, and *PLPP2* in patients who died during the follow-up (*P* < 0.05). *GCNT3* was overexpressed in tumors from deceased patients, but it was not statistically significant (*P* = 0.06)

We further explored the gene expression of the 4 identified genes in the TCGA-KIRC dataset to validate our findings (Supplementary Table 4). All 4 genes were overexpressed in advanced stage compared to early stage ccRCC (*P* < 0.05), and *G6PD*, *APLP1* and *GCNT3* were overexpressed in high grade compared to low grade ccRCC (*P* < 0.05). *G6PD*, *APLP1*, and *PLPP2* were overexpressed in ccRCC with necrosis (*P* < 0.05).

However, only *G6PD* was significantly over-expressed in tumor compared to normal tissues (*P* = 0.001). *PLPP2* gene was over-expressed in tumor, but was not statistically significant (*P* = 0.07). On the other hand, *APLP1* was over-expressed in normal compared to tumor samples (*P* < 0.001). When expression levels in normal samples were examined, significantly higher expression of *G6PD* and *PLPP2* was observed in patients with advanced stage ccRCC compared to early stage ccRCC (*P* < 0.05). No significant difference in gene expression was observed for grade and necrosis in normal tissues.

### Survival analysis with TCGA-KIRC dataset

**Figure 1:**
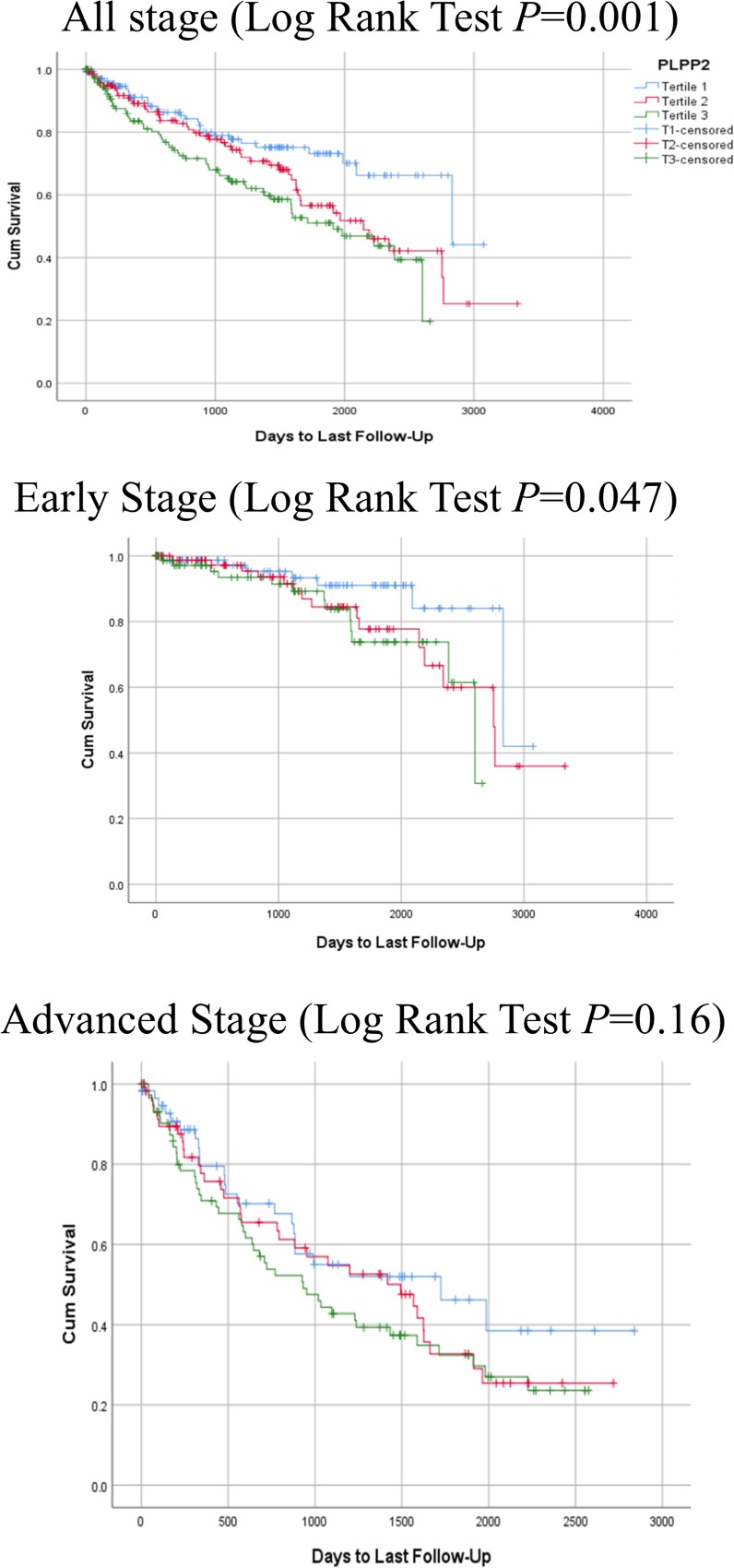
Kaplan-Meier plots for the *PLPP2* gene **A.** all stages (Log Rank Test *P* = 0.001), **B.** early stage (Log Rank Test *P* = 0.047), and **C.** advanced stage (Log Rank Test *P* = 0.16).

In an unadjusted model, expression of *APLP1*, *G6PD*, and *PLPP2* were significantly associated with OS in the TCGA-KIRC dataset (Supplementary Figure 3, Supplementary Table 5). When we stratified the patients by stage, *PLPP2* expression showed significant association with OS in the early stage group (Log Rank Test *P* = 0.047), but not in the advanced stage group (Figure 1). We did not see this pattern of differential expression for any of the other genes.

In Cox regression analysis, higher expression of *APLP1, G6PD* and *PLPP2* were associated with poorer OS in an unadjusted model (Quartile 1 *vs*. Quartile 4, HR = 2.78, 95% CI:1.65-4.68, HR = 1.99, 95% CI:1.26-3.15, and HR = 2.27, 95% CI:1.38-3.72, respectively, all *P* < 0.001). Once the model was adjusted, these associations were no longer significant. Further investigation stratifying by stage found that *APLP1* and *PLPP2* overexpression were significantly associated with poorer OS in the early stage (Quartile 1 *vs*. Quartile 4, HR = 3.87, 95% CI:1.25-11.97, *P* = 0.02 and HR = 4.77, 95% CI:1.37-16.57, *P* = 0.04 respectively) in an adjusted model. Linear trend of association was significant for *APLP1* expression (*P* = 0.006) and approaching significance for *PLPP2* expression (*P* = 0.06).

## DISCUSSION

Previous gene expression studies identified some biomarkers and signatures that are associated with aggressive ccRCC or ccRCC prognosis and survival [5–11]. However, these studies mainly included European populations. With over-representation of HAs, this study attempted to identify novel molecular signatures of aggressive ccRCC. It was in the analysis of the association of differential gene expression with SSIGN score that we made our most significant observations and found 4 genes that were over-expressed in tumors with high SSIGN score as well as advanced stage and high grade ccRCC and ccRCC with necrosis. Of 4 genes, 2 of them, *APLP1* and *PLPP2*, were also associated with OS in early stage ccRCC.

*APLP1*, involved in glucose homeostasis, was a novel finding showing strong association with OS in ccRCC but was over-expressed in normal compared to tumor samples (*P* < 0.001). *APLP1* is closely related to *APP* and *APLP2*, both of which are associated with aggressive cancers in other organs [22]. *PLPP2*, was over-expressed in advanced stage and high-grade tumors and tumors with necrosis, and was also associated with OS in patients with early stage ccRCC. *PLPP2* is a gene in the phosphatidic acid phosphatase (PAP) family and a key enzyme in lipid metabolism. Lipid Phosphate Phosphatases are likely to be involved in cancer cell proliferation [23]. Other genes that topped the list of differential expression were *G6PD* and *GCNT3*. Association between expression of *G6PD*, glucose-6-phosphate dehydrogenase, and aggressiveness was validated using the TCGA KIRC dataset. *G6PD* is an enzyme involved in carbohydrate metabolism, conversion of glucose to ribose-5-phosphate. The previous studies show that *G6PD* is involved in RCC proliferation and associated with survival [24, 25], and is a drug resistance gene in colon cancer [26]. Another gene, *GCNT3*, glucosaminyl (N-acetyl) transferase 3, is involved in colon and pancreatic cancer [27, 28].

Of these genes, only *G6PD* were validated with the TCGA dataset using our criteria. It is possible that it was due to small sample size in our study, but it should also be noted that discovery samples we used had different demographic characteristics than the TCGA data. In the TCGA data, a large majority of samples (82.7%) were individuals of European descent, while our discovery data composed of only 45.5% EAs. It is likely that HAs and other racial/ethnic groups have very different behavioral and social factors that contributed to tumor characteristics at diagnosis. For example, genes associated with metabolism, *G6PC*, *PLPP2*, and *APLP1*, were over-expressed in advanced stage and high grade ccRCC. Obesity is associated with higher kidney cancer mortality [29], and distribution of adiposity or body composition varies across racial/ethnic groups [30]. Obesity may affect ccRCC progression through different mechanisms in EAs and HAs. Obesity and other behavioral factors may also affect tumor microenvironment [31, 32], and *G6PD* and *PLPP2* were also overexpressed in normal tissues form patients with advanced stage ccRCC in the TCGA dataset. In a follow-up study, we will assess the relationships between obesity and molecular biomarkers in ccRCC progression. Besides these four genes, additional sets of genes associated with ccRCC progression in other studies were also associated with SSIGN score, providing a molecular signature for this clinical assessment score.

There are several major limitations of current study. First, small sample size was very small in the initial gene expression analysis, so we were not able to perform analysis adjusting for relevant variables. Also, we may have observed false positive signatures, which could not be verified using the TCGA data. Moreover, sample size of our discovery dataset did not allow us to fully investigate the causal relationship between gene expression signatures and ccRCC aggressiveness. To test the causal relationship, we used the TCGA KIRC dataset to test if expression of these genes is associated with OS. Of 4 genes identified, only *G6PD* was validated and *APLP1* and *PLPP2* were associated with OS. The difference between our discovery and the TCGA data set could result from the difference in sensitivity and specificity of the TempO-Seq platform compared to RNA-sequencing in the TCGA data, difference patient characteristics, and sample size. Lastly, it could also have been due to the physical differences in the samples used. We obtained 1 mm punches of FFPE with about 5 mm thickness, and assayed the entire sample. The TGCA data set used RNA/DNA co-extracted from 30mg of frozen surgical resections. Tumor tissue from a single patient can be very heterogeneous, such that using a large amount of sample may have impacted study findings. None-the-less, to develop a clinically useful test, it is necessary to test FFPE, which is one reason why we set out to demonstrate the ability to do so in this study. We demonstrated that the TempO-Seq assay from FFPE samples can identify previously reported gene expression signatures and novel signatures associated with high SSIGN score and OS. In the follow-up study, we will test with a larger sample to permit finer-scale molecular subtype analysis and race/ethnicity stratified analysis; 1) using 1 mm2 areas of >90% cancer tissue for assay; and 2) taking advantage of an advance in the TempO-Seq technology (Digital Spatial Molecular Profiling) which permits even smaller areas of tissue to be profiled and correlated directly to the morphology of the tissue.

## MATERIALS AND METHODS

### Samples

After Institutional Review Board approval, the clinical information of RCC patients, who underwent robotic, laparoscopic or open partial or radical nephrectomy at our University hospital between 2010 and 2016 were reviewed (*n* = 361), 51 samples were selected and reviewed by board certified genitourinary pathologists. The final set of 33 patients who were diagnosed with ccRCC and had no previous diagnosis of cancer were selected based on staging, with close to equal representation of HA and European American (EA) patients. Patients' demographic (age at surgery, gender, and race/ethnicity) and clinical information, including pre-operation creatinine levels, glomerular filtration rate (GFR), histological subtype, stage at diagnosis, Fuhrman Grade, tumor size, and presence of necrosis, was obtained from their electronic medical record. The Stage, Size, Grade, and Necrosis (SSIGN) score [3, 4], a composite score of these 4 clinical assessment measures, was used to define aggressiveness of ccRCC; non-aggressive (SSIGN ≤3), intermediate (SSIGN between 4 and 6), and aggressive (SSIGN≥7).

Corresponding Hematoxylin and Eosin (H&E) stained slides were retrieved for each patient identified, and each slide was reviewed by a board-certified pathologist and pathology resident. Slides were evaluated to identify areas of highest tumor grade of RCC, with morphologic grading as described by the International Society of Urological Pathologists (ISUP) and World Health Organization. The highest-grade regions were identified and marked on the slide. Additionally, areas of normal, uninvolved kidney parenchyma were also identified and marked. Corresponding FFPE blocks were then retrieved for both high-grade and non-neoplastic regions. The blocks were compared against the associated marked H&E stained slides and the corresponding regions were marked on the tissue blocks. 1 mm punches of tissue were then taken from the blocks.

### Whole transcriptome sequencing

Expression data for 21,111 transcripts were generated using TempO-Seq™. TempO-Seq was selected, because it does not require RNA extraction from the FFPE, only requires a PCR instrument and a sequencer, and comes with an automated data analysis package for processing the sequencing data and determining differential expression and subtype score, making this assay suitable for use in any research laboratory and in diagnostic and clinical pathology laboratories. TempO-Seq also has the ability to detect low quality and fragmented RNA typically found in FFPE samples [14, 15]. Briefly, 1mm diameter cores of FFPE renal cancer and adjacent normal tissue were added to a lysis reagent with mineral oil, and then heated at 80°C for 5 minutes to deparaffinize the tissue. Tissue was then digested with a protease until fully lysed, then heated to 95°C for 15 min (note that the commercial FFPE kits use a slightly different protocol). A fraction of this lysate was added to a mixture of TempO-Seq whole transcriptome Detector Oligos (DOs) which were then hybridized, cleaned up with an exonuclease, and then ligated. The samples were then amplified via PCR using barcoded primers and then pooled into a sequencing library. All reagents were from a pre-release TempO-Seq FFPE assay kit, now commercially available. The sequencing libraries were purified using the Clontech NucleoSpin Gel and PCR Cleanup Kit (Clontech), and sequenced using an Illumina NextSeq, to generate demultiplexed FASTQ files. The libraries were compatible with sequencing on the Illumina platform. Automated TempO-SeqR software was used to align the demultiplexed FASTQ files and produce a read count table for all the samples in the sequencing run, and for other steps, including normalization, averaging of replicates, and calculating averages and statistics for technical replicates without need for expert bioinformatics analysis of the sequencer output.

### Statistical analysis

Independent sample T-test for normally distributed continuous variables, Mann-Whitney U-test for variables that deviate from normality, and chi-squared test were used to understand patients' characteristics. We evaluated if the samples clustered into ccA or ccB using the set of genes identified by Brannon et al [6]. Because of the differences in gene expression platform used by Brannon (microarray) and the targeted sequencing TempO-Seq assay, and differences introduced by using purified RNA for microarrays *vs* directly assaying lysed FFPE in the TempO-Seq assay, we could not simply compare the expression levels of the signature genes. Instead, a ccA/ccB score was calculated using a modified algorithm which assigned each sample an A/B score based on whether each gene from the panel was over/under-expressed compared to the average expression for each sample. The final score was the weighted average of the over-expression of group A divided by the weighted average of group B using the coefficients from Brannon et al. Even though of the 103 genes in the Brannon A *vs* B signature, 85 genes were not differentially expressed in the TempO-Seq data, we kept these in the ratio calculation used to assign subtype. Chi-squared tests were used to test the association between molecular subtypes and race/ethnicity as well as between molecular subtypes and pathological characteristics, including SSIGN, and independently, stage, grade, performance status, and necrosis. Association between molecular subtypes and tumor size was tested using the Mann-Whitney U test.

To identify a gene signature associated with outcomes predicted by the SSIGN score, gene expression in aggressive ccRCC (SSIGN score ≥7) was compared to that of non-aggressive ccRCC (SSIGN ≤3). Then, the result was compared to results of differential gene expression analysis for individual clinical characteristics: 1) Fuhrman Grade of 3 or 4 *vs*. 1 or 2; 2) Stage III or IV *vs*. I or II; and 3) necrosis present *vs*. absent. The DESeq2 differential expression algorithm was used to identify differentially expressed genes [16], and Benjamini and Hochberg's procedure was used to adjust for multiple testing [17]. We considered *P*-values less than 0.05 after adjusting for multiple testing (*P*_ADJ_) as statistical significant. After log_2_-transformation, Mann-Whitney U-test and Spearman's correlation was used to evaluate if the top identified genes are correlated with clinical characteristics and outcomes (recurrence and mortality).

TCGA-KIRC level 3 RNA-Seq Upper Quartile normalized FPKM data for ccRCC (451 patients without previous diagnosis of cancer) were used for validation of our findings and to assess associations between expression of identified genes and OS. After log_2_ transformation, related-samples Wilcoxon Signed Rank Test was used to test if the identified genes were differentially expressed between tumor and normal tissues, and independent samples Mann-Whitney U-test was used to test if identified genes were over-expressed or down-regulated in advanced stage and high-grade tumors. The criteria for validation are differentially expressed between tumor and normal tissues and over-expressed or down-regulated in advanced or high-grade tumors or tumors with necrosis. We considered *P* < 0.05 as significant for validation. Survival analysis was also performed using TCGA data. Survival curve was plotted using Kaplan-Meier method, and Log Rank Test as well as Cox regression was used to assess if expression of identified genes predicts OS adjusting for age, gender, race/ethnicity, stage, and grade. Linear trend was examined using the median gene expression of each quartile as a continuous variable.

## CONCLUSION

We demonstrated that an extraction-free transcriptomics assay of FFPE was able to detect existing as well as potentially novel expressed gene biomarkers associated with more aggressive ccRCC. In the current study, only 33 patients were included for discovery of novel gene expression signatures. However, inclusion of a previously underrepresented population in this study identified unique biomarkers that have not been observed or well characterized in previous gene expression studies of ccRCC. Patients with molecular biomarkers of aggressiveness may be counseled towards radical nephrectomy over nephron sparing surgery.

## SUPPLEMENTARY MATERIALS FIGURES AND TABLES


