# Supplementary material for: Whole-transcriptome sequencing identified gene expression signatures associated with aggressive clear cell renal cell carcinoma
Source: Genes Cancer. 2018 May;9(5-6):247–56. doi: 10.18632/genesandcancer.183 (PMC6305109; doi:10.18632/genesandcancer.183)
Supplement: Supplementary file 1 [file ganc-09-247-s001.pdf]

Whole-transcriptome sequencing identified gene expression signatures associated with aggressive clear cell renal cell carcinoma – Batai et al

Supplementary Table 1: Expression difference (log_2 Fold Change) of genes implicated in RCC between aggressive (SSIGN ≥7) and non-aggressive (SSIGN ≤3) tumors.

Gene	log2FC	*P*	P_{ADJ}
BAP1	-0.88	0.048	0.33
SETD2	-0.93	0.048	0.33
PBRM1	-0.53	0.20	0.63
KDM5C	0.23	0.30	0.73
VHL	-0.31	0.34	0.77
PTEN	0.14	0.79	0.96
MTOR	0.09	0.79	0.96

Supplementary Table 2: Differential expression (log_2 Fold Change) of previously reported genes in aggressive (SSIGN ≥7) and non-aggressive (SSIGN ≤3) tumors.

Gene	log2FC	*P*	P_{ADJ}	References
S1PR1	-1.99	8.30E-06	0.002	Wozniak et al. 2013
TSPAN7	-2.25	9.36E-06	0.002	Thibodeau et al. 2016
RGS5	-2.17	1.64E-05	0.003	Yao et al. 2008
TEK	-2.12	3.54E-05	0.004	Kosari et al. 2005
ERG	-1.79	4.39E-05	0.005	Kosari et al. 2005
PTPRB	-1.92	6.06E-05	0.006	Kosari et al. 2005
CYYR1	-1.93	7.55E-05	0.007	Wozniak et al. 2013
TMTC1 (ARG99)	-1.83	1.00E-04	0.008	Kosari et al. 2005
HK2	1.69	1.00E-04	0.009	Thibodeau et al. 2016
SYNPO2	-1.52	1.00E-04	0.01	Kosari et al. 2005
LDB2	-1.86	1.40E-04	0.01	Kosari et al. 2005; Wozniak et al. 2013
CLEC14A	-1.82	1.69E-04	0.01	Wozniak et al. 2013
EPAS1	-1.53	3.00E-04	0.02	Kosari et al. 2005
CLEC1A	-1.6	2.80E-04	0.02	Wozniak et al. 2013
EDNRB	-1.68	6.70E-04	0.03	Yao et al. 2008
CKS2	1.15	9.00E-04	0.03	Kosari et al. 2005

SPP1	1.28	0.002	0.046	Cheng et al. 2017
LOX	1.52	0.002	0.047	Thibodeau et al. 2016
PLPP3 (PPAP2B)	-1.15	0.002	0.049	Yao et al. 2008
NPY1R	-1.44	0.002	0.052	Kosari et al. 2005
FILIP1	-1.47	0.002	0.055	Kosari et al. 2005
TMEM204	-1.37	0.002	0.058	Wozniak et al. 2013
C5orf46	1.64	0.004	0.08	Thibodeau et al. 2016
SDPR	-1.62	0.0049	0.09	Kosari et al. 2005

Supplementary Table 3: Correlation between gene expression and clinical outcomes (recurrence and mortality)

	**Median Log_2 Expression (Interquartile Range)**		***P***
	Recurrence* (No, n=22)	Recurrence* (Yes, n=4)	
APLP1	5.31 (4.06-5.79)	7.64 (5.60-10.62)	0.03
G6PD	9.51 (8.88-9.90)	10.88 (10.77-11.60)	0.01
GCNT3	4.03 (2.84-5.22)	4.35 (4.18-6.14)	0.25
PLPP2	5.39 (4.03-6.34)	6.28 (5.12-9.32)	0.20
	Alive (n=29)	Deceased (n=4)	
APLP1	5.35 (4.49-6.36)	8.63 (6.67-10.62)	0.02
G6PD	9.60 (8.96-10.78)	11.89 (11.18-13.07)	0.009
GCNT3	4.10 (3.28-5.51)	6.13 (4.63-8.46)	0.06
PLPP2	5.53 (4.34-6.60)	8.65 (6.98-9.96)	0.006

* Analysis for recurrence does not include patients with metastatic ccRCC.

Supplementary Table 4: TCGA validation study

	Median Log Expression		*P*
**Tumor**			
	Early Stage (I and II)	Advanced Stage (III and IV)	
APLP1	13.26	13.74	<0.001
G6PD	17.60	17.89	<0.001
GCNT3	15.12	15.40	<0.001
PLPP2	14.02	14.46	<0.001
	Low Grade (1 and 2)	High Grade (3 and 4)	
APLP1	13.28	13.66	<0.001
G6PD	17.53	17.87	<0.001
GCNT3	15.00	15.50	<0.001
PLPP2	14.04	14.39	0.12
	Necrosis (No)	Necrosis (Yes)	
APLP1	13.81	13.36	0.004
G6PD	17.99	17.67	<0.001
GCNT3	15.47	15.20	0.053
PLPP2	15.32	14.15	0.009
**Normal**			
	Early Stage (I and II)	Advanced Stage (III and IV)	
APLP1	14.87	14.72	0.88
G6PD	17.50	17.62	0.003
GCNT3	15.12	15.99	0.10
PLPP2	13.47	14.23	<0.001
	Low Grade (1 and 2)	High Grade (3 and 4)	
APLP1	14.83	14.96	0.31
G6PD	17.54	17.60	0.08
GCNT3	15.57	15.61	0.61
PLPP2	13.65	14.07	0.21
	Necrosis (No)	Necrosis (Yes)	
APLP1	14.87	15.11	0.44
G6PD	17.55	17.68	0.13
GCNT3	15.57	15.50	0.75
PLPP2	13.85	13.92	0.68

Supplementary Table 5: Results of Cox Regression analysis for overall survival using TCGA data

	Unadjusted HR (95% C.I.)	P_{Trend}	Adjusted HR (95% C.I.)	P_{Trend}
**All Stages**[1]				
APLP1				
Quartile 1	Reference	<0.001	Reference	0.23
Quartile 2	1.61 (0.92-2.81)		1.35 (0.76-2.40)	
Quartile 3	2.35 (1.38-4.00)		1.95 (1.13-3.37)	
Quartile 4	2.78 (1.65-4.68)		1.44 (0.83-2.50)	
G6PD				
Quartile 1	Reference	<0.001	Reference	0.16
Quartile 2	0.93 (0.54-1.61)		0.82 (0.47-1.43)	
Quartile 3	1.28 (0.77-2.14)		1.09 (0.64-1.87)	
Quartile 4	1.99 (1.26-3.15)		1.42 (0.88-2.30)	
GCNT3				
Quartile 1	Reference	0.33	Reference	0.49
Quartile 2	0.68 (0.40-1.15)		0.69 (0.40-1.18)	
Quartile 3	1.07 (0.67-1.71)		0.81 (0.49-1.33)	
Quartile 4	1.14 (0.72-1.80)		0.79 (0.48-1.30)	
PLPP2				
Quartile 1	Reference	0.004	Reference	0.25
Quartile 2	1.58 (0.93-2.67)		1.38 (0.81-2.36)	
Quartile 3	1.16 (0.67-2.01)		1.04 (0.60-1.82)	
Quartile 4	2.27 (1.38-3.72)		1.45 (0.88-2.43)	
**Early Stage (I and II)**[2]				
APLP1				
Quartile 1	Reference	0.02	Reference	0.006
Quartile 2	0.89 (0.32-2.48)		0.65 (0.21-2.04)	
Quartile 3	2.52 (1.00-6.37)		2.48 (0.87-7.10)	
Quartile 4	2.70 (0.99-7.38)		3.87 (1.25-11.97)	
G6PD				
Quartile 1	Reference	0.68	Reference	0.31
Quartile 2	0.99 (0.42-2.33)		0.95 (0.38-2.39)	
Quartile 3	0.61 (0.19-1.92)		0.91 (0.28-3.04)	
Quartile 4	1.25 (0.53-2.95)		1.58 (0.60-4.18)	
GCNT3			b	
Quartile 1	Reference	0.97	Reference	0.90
Quartile 2	0.86 (0.32-2.30)		1.18 (0.42-3.34)	
Quartile 3	1.05 (0.41-2.66)		1.12 (0.39-3.20)	
Quartile 4	0.97 (0.37-2.52)		0.96 (0.35-2.67)	
PLPP2				
Quartile 1	Reference	0.02	Reference	0.06
Quartile 2	3.53 (1.14-10.97)		3.63 (1.05-12.65)	

Quartile 3	1.71 (0.50-5.86)		1.66 (0.44-6.22)	
Quartile 4	5.20 (1.64-16.46)		4.77 (1.37-16.57)	
**Advanced Stage (III and IV)**[2]				
APLP1				
Quartile 1	Reference	0.35	Reference	0.74
Quartile 2	1.60 (0.81-3.17)		1.38 (0.69-2.79)	
Quartile 3	1.91 (0.99-3.68)		1.61 (0.82-3.15)	
Quartile	1.54 (0.82-2.89)		1.27 (0.66-2.44)	
G6PD				
Quartile 1	Reference	0.10	Reference	0.18
Quartile 2	0.84 (0.42-1.71)		1.06 (0.51-2.20)	
Quartile 3	0.93 (0.51-1.71)		0.91 (0.49-1.69)	
Quartile 4	1.37 (0.78-2.40)		1.37 (0.77-2.43)	
GCNT3				
Quartile 1	Reference	0.54	Reference	0.84
Quartile 2	0.68 (0.36-1.27)		0.64 (0.33-1.21)	
Quartile 3	1.04 (0.61-1.79)		0.85 (0.48-1.50)	
Quartile 4	1.08 (0.64-1.81)		0.87 (0.49-1.54)	
PLPP2				
Quartile 1	Reference	0.42	Reference	0.85
Quartile 2	1.17 (0.64-2.15)		1.16 (0.62-2.14)	
Quartile 3	0.94 (0.51-1.74)		0.95 (0.51-1.80)	
Quartile 4	1.31 (0.75-2.27)		1.11 (0.63-1.96)	

[1] Adjusted for age, gender, race/ethnicity, stage, and grade.

[2] Adjusted for age, gender, race/ethnicity, and grade

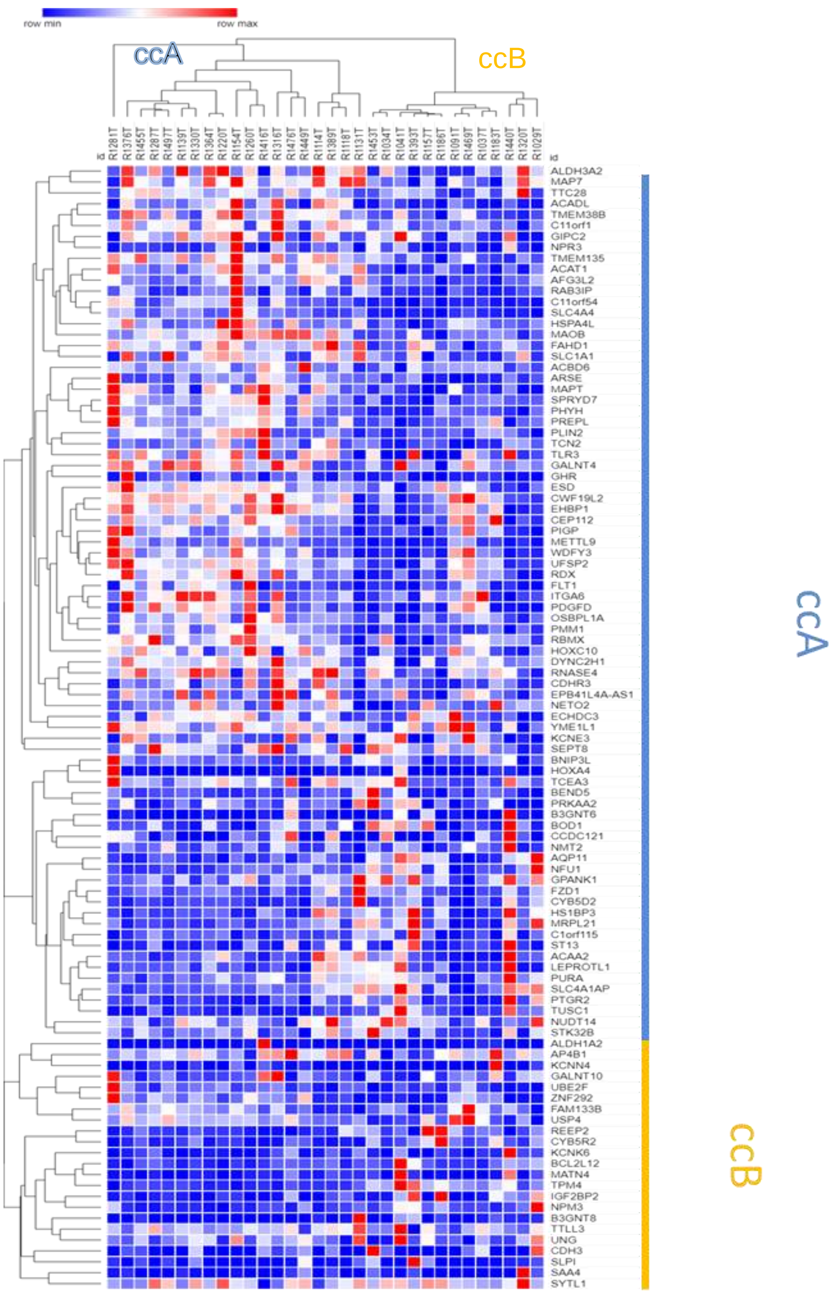

Supplementary Figure 1: Heatmap and cluster plot showing clustering of molecular subtype ccA and ccB

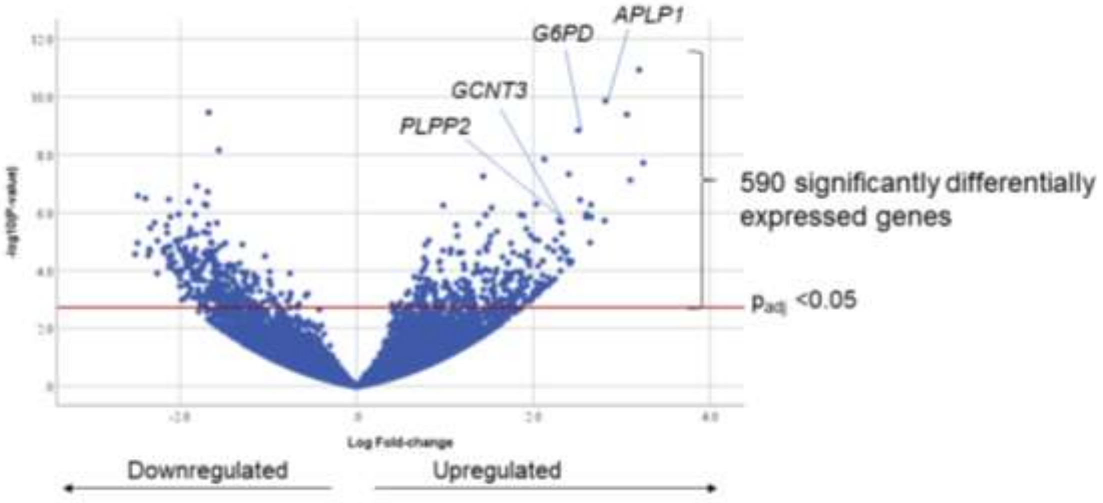

Supplementary Figure 2: Volcano plot showing 590 significantly differentially expressed genes between high (≥7) and low SSIGN (≤3), and the four genes that were also over-expressed in advanced stage and high grade ccRCC as well as ccRCC with necrosis

A. *APLP1*

All stage (Log Rank Test *P*=0.007)

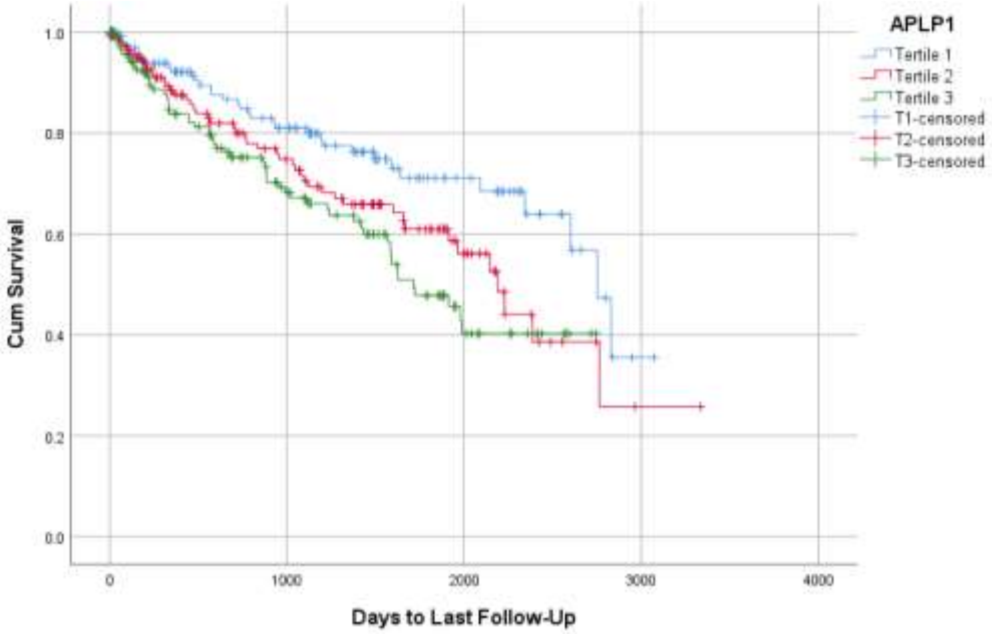

Early Stage (Log Rank Test *P*=0.42)

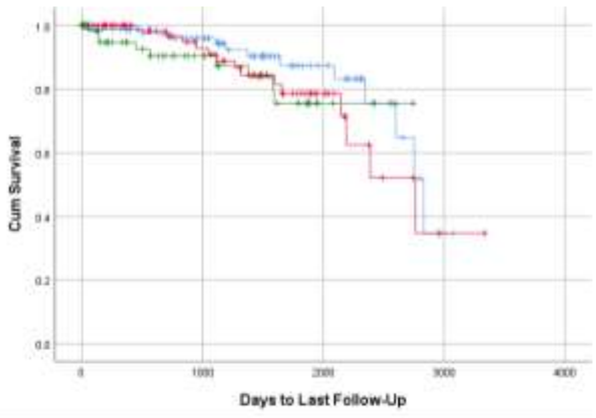

Advanced Stage (Log Rank Test *P*=0.60)

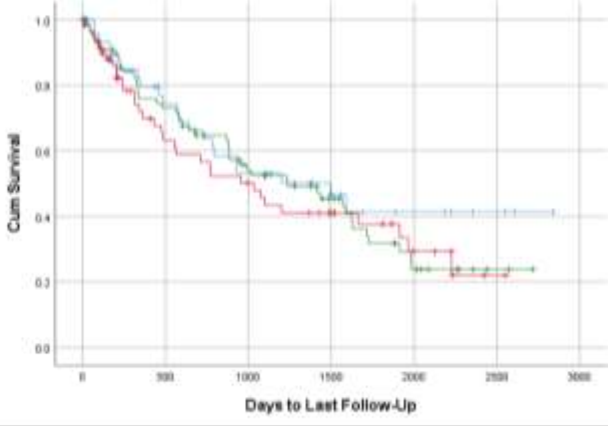

B. *G6PD*

All stage (Log Rank Test *P*=0.007)

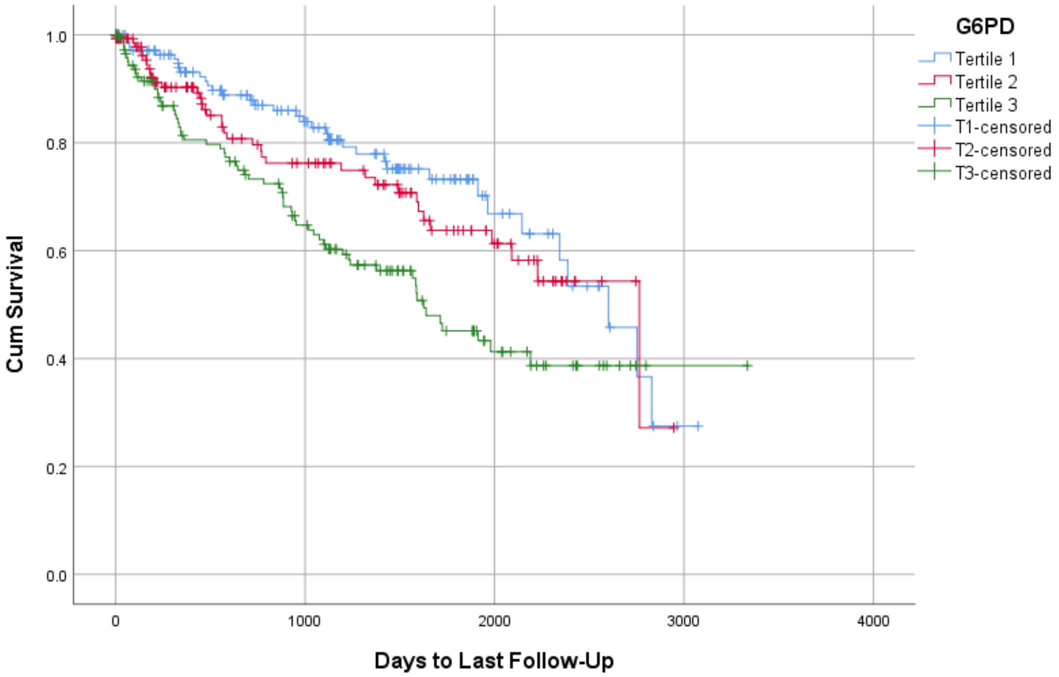

Early Stage (Log Rank Test *P*=0.65)

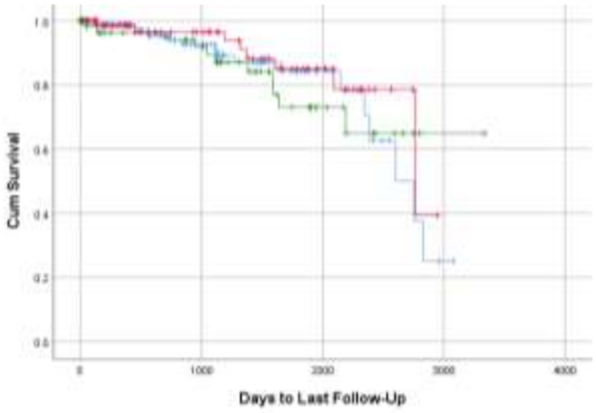

Advanced Stage (Log Rank Test *P*=0.35)

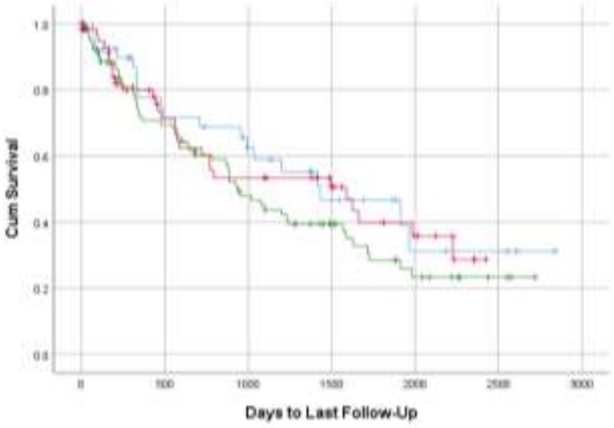

C. *GCNT3*

All stage (Log Rank Test *P*=0.57)

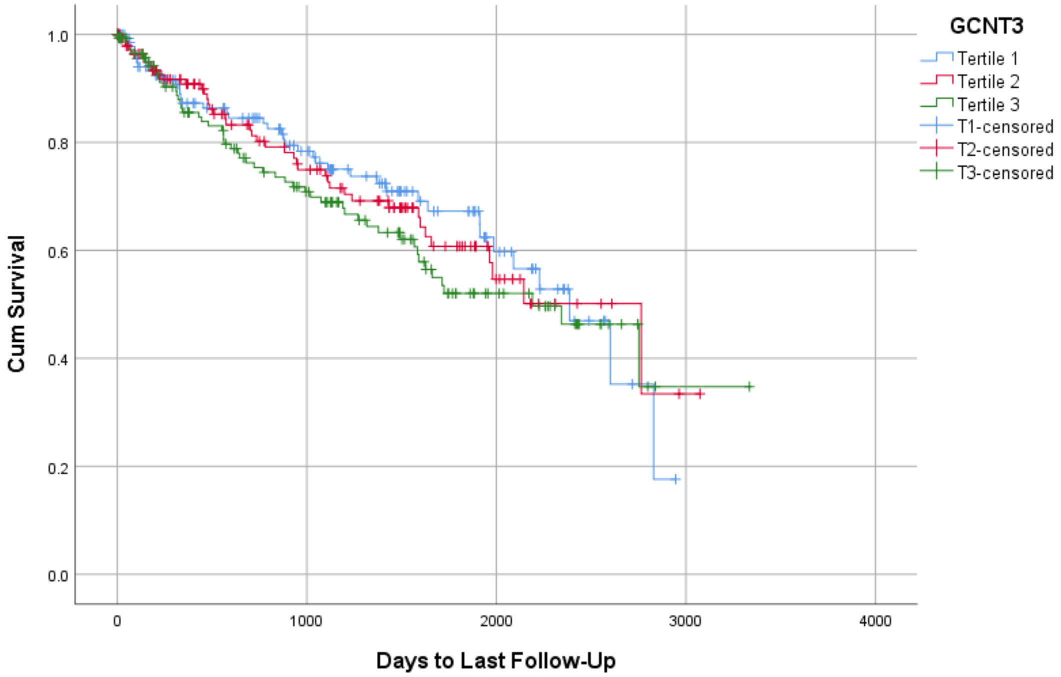

Early Stage (Log Rank Test *P*=0.89)

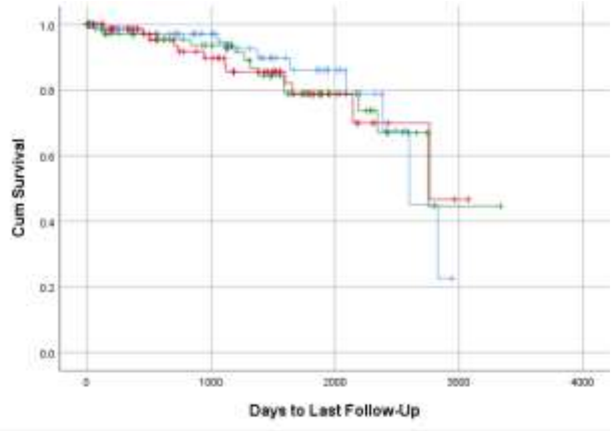

Advanced Stage (Log Rank Test *P*=0.63)

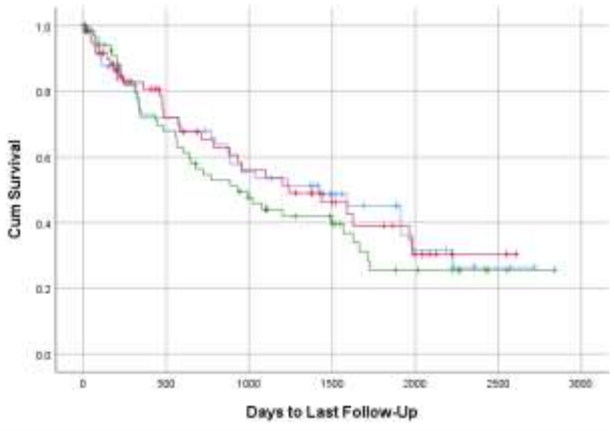

Supplementary Figure 3: Kaplan-Meier Plots for *APLP1*, *G6PD*, and *GCNT3*
